# Hereditary Transmission and Variation by Descent

**Published:** 1875-06

**Authors:** 


Article II.

### HEREDITARY TRANSMISSION AND VARIATION BY DESCENT.

Dr. Brown-Sequard, a gentleman who is a foster-parent of physiological science, but a terrible bane to guinea pigs, says, from a late series of experiments upon these little animals, that injuries to the parents result in analogous lesions in the children even to the extent that a young pig is born without toes, of parents from whom the toes have been removed artificially. Some of his conclusions concerning the hereditary transmision of morbid states to many animals, caused in either one or both of their parents by some injury to the nervous system, are as follows:

1. A change in the shape of the ear, of those animals in which such a change was caused by a division of the cervical sympathetic nerve in the parent.

2. Partial closure of the eyelids, in animals born of parents in which that state of the eyelids had been caused, either by section of the cervical sympathetic nerve, or by removal of the superior cervical ganglion.

3. Dry gangrene of the ears, in animals born of parents in which these ear alterations had been caused by injury to the restiform body.

4. Section of the sciatic nerve has been practiced in animals, with the result of rendering portions of the foot anæsthetic, which portion was eaten off by the animal, and the result was the production of the same deformity in its offspring for three generations; thus showing that the sciatic nerve acquired the inherited power, in the congenitally toeless animal, of transmitting this peculiar deformity.

The law of hereditary variation in species, of plants, animals and man, is the cause of their respective varieties. Every organism bears a general resemblance to its parents, but it is equally true that no organization is exactly like its parent—it differs in numerous traits of more or less importance. No two plants are alike, and no two animals exist without difference. It is almost incomprehensible what variation there is in human beings. It is strange, indeed, that there can be such differences in the human face, and yet so many be considered good looking.

Charles Darwin, who has immortalized himself—and the monkey—has probably made more investigations on the subject of variation of species than any other man. His experiments were made upon pigeons. Beginning with a pair of the original stock—the rock pigeon—he succeeded, by his favorite and celebrated methods of "Sexual selection" and "Survival of the fittest," in producing about one hundred and fifty varieties of pigeons.

Another instance in point was the Ancon sheep, a variety at one time well known in this country. The stock originated in 1791, in a flock of sheep consisting of one male and thirteen females, owned by Seth Wright, a farmer in Mass. Of this flock a lamb was born of very peculiar formation. It had a very long body, very short legs, and the legs were bowed. The lamb was a male, and Wright, anticipating Darwin, bred from this male, and in time produced a flock of an entirely new variety of sheep. The variety was, however, lost afterward by mixture with other varieties. Darwin concludes, therefore, that all the varieties of plants, animals and man originate in the great law of hereditary transmission and variation by descent. Regarding the human species, therefore, I am not one of those who can believe otherwise than that all varieties sprang originally from a single pair, nor can I see any good ground whatever, or any tenable sort of evidence, for believing that there is but one species of man. Nevertheless, as there are great numbers and varieties of plants and animals, so are there many remarkable varieties of men. I speak not particularly of the distinctions in races, but there are some most astonishing varieties in men of the same race, in regard to structural development, especially to the structural development of that dominant organ, brains. In the human species, also, the transmission of abnormal structures occurs. A very notable instance was that of a Maltese, named Gratio, who was born with six fingers on each hand. He married a woman with the usual number of fingers, and they had four children, two of whom had the same deformity. This deformity was lost after the third generation. It is plain to be seen that had members of this family intermarried, there would have been a six-fingered variety of mankind, but their "sexual selection" was under their own control, and the peculiarity was swallowed up in pure blood, or, more strictly, pure nervous influence. Cleft palate, club foot, and kindred morbid variations and vagaries of human generation, depend upon the same cause. When Darwin can take a pair of monkeys and pen them up, and by controlling their sexual selection, nursing their varieties, killing the weakest, so as to allow the survival of the fittest, and bring out a new Adam and Eve as the result of his breeding, faith will increase in his doctrines.

Hereditary transmission of disease in human subjects—our patients—is subject to the same rules as variation of the species. Brown-Sequard, in the course of his investigations into the causes of epilepsy, discovered a method by which epilepsy could be originated. Guinea pigs were of course the victims upon which he operated, and eventually he discovered the remarkable fact, that the young of those animals upon which he had produced epilepsy, were epileptic. The artificially produced epilepsy in the parents, caused constitutional epilepsy in the young. Here then we have an instance, which, standing alone, decides the question. We have a special form of nervous disease, not caused by any natural variation of structure or of function that has arisen spontaneously in the organism, or caused directly to the nerves by mechanical injury. There is the special form of nervous aberration confirmed by repetition. The fits are more readily and easily induced, until there is established the epileptic habit, that is, the various actions constituting the fit produce in the nervous system such structural changes, that result in repeated epilepsy, and affecting the reproductive centres, cause them to produce organisms with the same epileptic tendencies. Statistics show that one-half of the epileptic cases are directly traceable to inherited tendency.

Scrofula and syphilis are types of hereditary diseases. Their laws of transmission are well understood by the medical profession, and are subject, in cause, origin and transmission, to the same laws as the transmission of variation in species. They *are* simply the variations of the species of human beings, that we term disease, because they are negative variations, and the tendency is to dissolution of the organism.

There is another property of hereditary transmission which I will now bring forward, called atavism. It is taken from a word meaning ancestor, and means that peculiar ancestral traits may skip over a generation or two, and then reappear. It also refers to the tendency in species to preserve their integrity against variation. In medicine, it covers the ground expressed by "Vis medicatrix naturæ." One is often surprised to see a black lamb, or one with black spots, in a flock of white sheep, but generally his darkness can be traced to his colored ancestral parent. Instances still more remarkable, in which the remoteness of the ancestors is very great indeed, are quoted by Darwin. He reports that in crosses between varieties of the pigeon, there will sometimes reappear the plumage of the original rock pigeon, from which these varieties descended. By atavism, varieties of species are sometimes lost—they recover the original form of the species from which the variation occurred, as in the instance of the Ancon sheep. Instances have been known in the human family, generally those inhabiting the sunny South, where a black baby has suddenly and unwelcomely made his appearance and paled his white ancestors with terror. In such cases, the peace of families is *sometimes* restored by the resurrection of the long-buried secret, that away back among the blooded ancestry, there had been a little unfortunate miscegenation. By means of atavism, or dilution with pure blood, human sins are washed away, as well as the vagaries and variations of human genesis. Diseases are only kept from engulfing the world, as the Asiatic deluge, by the mixture of pure blood as well as—the skill of the physician.

King David may have had a glimmering inspiration of this idea, when in his tottering age he took unto himself a young and blushing bride, that he might rejuvenate himself, but David was duly "gathered to his fathers."

The causes of variation in heredity. These are difficult to define, but may be enumerated as the external, internal, and spontaneous. Some cases of the external, as the operations of Sequard and Darwin, are well enough understood. Doubtless in nature the external causes are similar. The influence of climate, temperature, food, air, light, and the struggle for existence, have had the most to do with the variation and development of all species and their diseases in plants, animals and man.

As an instance of the influence of climate and social conditions upon the human species, I will refer you to one of the ruling elements of our society and body politic, the Irishman. This race, in this country, is an illustration of what external causes of variation can do. The difference between the general appearance, and mental and moral idiosyncrasies of the "Paddy from Cork" as he treads the bogs of his native "green isle of the sea" or appears in Castle Garden, N. Y., on landing from the emigrant ship, and his grandchildren, born and educated in America, is truly marvelous. The third generation of Irish in this country, is completely Americanized, in fact they are, in a large proportion, the Americans of to-day. The Irishman when he emigrates leaves his "Donnybrook fair," and generally his "shillalah," behind him, and, in the second generation, the "rich brogue" and many other Irish traits disappear, and the third generation is born American indeed and in truth. The Irishman in America must, by force of external causes, be wholly, and only, American.

The influence of light in variation of species, is beautifully illustrated in the eyeless fish of the great Mammoth Cave of Kentucky. The ancestors of these fish were undoubtedly at some time, by some means, introduced from without into the cave, and had eyes, but, being unable to escape and being in total darkness, there was no necessity for eyes, and as generation after generation was produced, the organs of sight gradually and entirely disappeared.

In view of these facts, the question would not seem to be, what variation in species *can* external causes produce, but what can they *not* produce.

I will only hint at the internal causes, for it implies too much biological knowledge to explain fully and to understand. By saying that every organism to exist must retain a due balance between its internal organic life forces, and those forces to which it is subject externally, it will be seen that this due balance may not always be adjusted perfectly, and in fact seldom is, and may be so far differentiated as to cause error of nutrition, disease, or some other variation, by disturbance of internal forces.

By spontaneous cause, is not meant exactly chance cause, but in reality it means that very little is known about it. Spontaneous, in science, and etiology, in medicine, means that the scientist and the physician don't like to say, I don't know—and so say, spontaneous. Perhaps a good illustration of these causes combined, may be found in the action of the mother's mind upon some peculiarities of the structural formation of her unborn child. Scientists and physicians have labored enthusiastically over what they term, or would term, if they could find it, spontaneous generation. They have hunted, as the alchemists hunted, for the elixir of life and the philosopher's stone, while the theologian has looked over their shoulders with bated breath, but with the smile of derision ready. One enthusiast watched an hermetically sealed tube containing a little grass and water, sixteen hours, through his microscope, for the bacteria which, like the old woman's soap, wouldn't come. The question may be asked, Where is the seat, or the immediate influence exerted that produces variation? Undoubtedly it is in the nervous system; whether the cause be external, internal, or spontaneous, its influence *must* be upon the nervous system, and there the impression is made that causes variation in the species, structural or pathological. Brown-Sequard's cases prove this. It is a hard blow to humoral pathology. The nervous system receives all impressions, and it is only the nervous system that can remember them and cause them to reappear, and so faithfully transmit them.

Now, then, how are variations transmitted? There is such a thing as a physiological unit. It bears the same relation to organic life that the unit or atom of matter does to the material universe, or that the number one does to mathematical science. The unit of matter, the atom, contains all the properties of the kind of matter to which it belongs. The unit of numbers contains all the properties of mathematics. The simplest form of figures may be found upon the schoolboy's slate, and perhaps a few upon the page of the physician's daily pocket record, but the combinations of these figures solve the problems of Euclid, and measure the distances between the fixed stars. The physiological unit, or the primal element of life, which men have so long looked to see spontaneously generated, contains all the properties of organic life. Its simplest form is the bacteria, the algæ, and the cell-life that we see coloring with a green tinge the surface of ponds of water. Its highest form is the illustrious ego, or man himself.

In generation and transmission, the germ-cell, and the spermatic-cell, are simply the vehicles, or common carriers of the physiological unit. The units meet, each carrying in its nature and constitution the heredity, the variations, the disease of the parent of the new organism about to be created. It is nature's most wonderful process. The units blend, unite, perhaps some variations of opposite character in the two units are cancelled, and here is one of nature's methods in atavism, new forces by their union are generated, and the result is, a new organism begins its development, with its character to a great extent predestined, as well as its heredity, by the physiological unit. We do not suppose that Calvin thought of this when he taught his doctrine of predestination, but for a man who was hunting in the dark, he came very near the truth. The two great contending elements in social life, science and religion, can be reconciled, if both will kneel humbly and learn of the physiological unit.

Gentlemen, to paraphrase an old saying: Take care of the units and the organisms will take care of themselves. Physiologists and scientists have satisfied themselves that the mind of man, his intelligence, his consciousness and moral nature depend upon the structure and physiological action of his brain. We are conscious living beings, only when the brain is in action. The existence of a separate entity, the soul, or spirit, operating through the brain, is believed by the greater portion of mankind, but it is certain, from observation, that man's mental status and moral nature are hereditary, and subject to the same laws as variation in every other respect. Physiologists have demonstrated that the gray cerebral cells of the cerebrum in action, generate the phenomena of thought and consciousness. Then mind, being directly one of the results of the combined action of the nervous system, what is so likely to be transmitted as mental peculiarities? The criminal beget criminal, the insane beget insane, as well as the gouty and scrofulous beget their kind. Religion may stand in the relation of a physician to the perverted, diseased moral nature, but to trace the analogy farther, there are so many quack religions in the world, that, though many are called, few come, or are chosen—that is, are cured.

Civilized nations have always had the care of the insane, and the criminal varieties of the human species, and have suffered thereby as individual organisms have by disease. It will probably always continue to be so, for there will probably never be mixture of pure blood enough to destroy the varieties by atavism, and the good will not bribe legislatures to condemn the wicked, the diseased, the monstrous, to eternal celibacy, and if they did, some of them would undoubtedly occasionally jump the fence, so to speak, and propagate their special variety by hereditary transmission and variation by descent.

